# Current Research on Antiepileptic Compounds

**DOI:** 10.3390/molecules201119714

**Published:** 2015-11-20

**Authors:** Cheng-Xi Wei, Ming Bian, Guo-Hua Gong

**Affiliations:** 1 Medicinal Chemistry and Pharmacology Institute, Inner Mongolia University for the Nationalities, Tongliao 028000, China; weichengxi1224@163.com (C.-X.W.); xiaopang1224@126.com (M.B.); 2 Affiliated Hospital of Inner Mongolia University for Nationalities, Tongliao 028000, Inner Mongolia, China

**Keywords:** epilepsy, antiepileptic compounds, structure activity relationship

## Abstract

Epilepsy affects about 1% of the world’s population. Due to the fact all antiepileptic drugs (AEDs) have some undesirable side effects and about 30% of epileptic patients are not seizure-free with the existing AEDs, there is still an urgent need for the development of more effective and safer AEDs. Based on our research work on antiepileptic compounds and other references in recent years, this review covers the reported work on antiepileptic compounds which are classified according to their structures. This review summarized 244 significant anticonvulsant compounds which are classified by functional groups according to the animal model data, although there are some limitations in the data. This review highlights the properties of new compounds endowed with promising antiepileptic properties, which may be proven to be more effective and selective, and possibly free of unwanted side effects. The reviewed compounds represent an interesting possibility to overcome refractory seizures and to reduce the percentage of patients with a poor response to drug therapy.

## 1. Introduction

Epilepsy is one of the more common and frequent neurological disorders in man, characterized by excessive temporary neuronal discharges resulting in uncontrolled convulsions that affect more than 2 million Americans and 60 million people worldwide [1]. If not treated, it is associated with progressively impaired cognition and function, brain damage, and other neurologic deficits. Although in many cases, epilepsy can be adequately controlled through administration of antiepileptic drugs (AEDs), it is estimated that roughly 20%–30% of patients have seizures that are resistant to available medical therapies [2,3,4].

Conventional AEDs like phenobarbital, primidone, phenytoin, carbamazepine, ethosuximide and the benzodiazepines are widely used. All currently approved antiepileptic drugs have dose-related toxicity and idiosyncratic side effects [5,6,7,8,9,10,11]. Therefore, the search for a newer, more effective, more selective agent with lesser side effects continues to be an area of investigation of medicinal chemists worldwide.

Anticonvulsant activities of new synthesized compounds were evaluated according to the Antiepileptic Drug Development Program of the National Institutes of Health (NIH) with the maximal electroshock (MES) test, the subcutaneous pentylenetetrazol (*sc*-PTZ) test, and the neurotoxicity was evaluated by the rotarod neurotoxicity test.

## 2. The Quinoline Functional Group

Quinoline is nitrogen-containing heterocyclic aromatic compound. Pharmacologically active substances display a broad range of biological activity. Quinoline has been found to possess anti-malarial, antibacterial, antifungal, anthelmintic, cardiotonic, anticonvulsant, anti-inflammatory, and analgesic activity. Our laboratory has studied a lot of quinoline derivatives for antiepileptic activity [12,13,14,15,16,17,18,19,20,21,22,23].

Xie *et al.*, reported a new series of 7-alkoxyl-4,5-dihydro-[1,2,4]triazolo[4,3-*a*]quinoline derivatives. Their anticonvulsant activities were evaluated by the MES test and the *sc*-PTZ test, and their neurotoxicity was evaluated by the rotarod neurotoxicity test with a median toxic dose (TD_50_) value of 54.5 mg/kg, MES and *sc*-PTZ tests showed that compound **1** (Table 1) was the most potent of this series with an effective dose (ED_50_) value of 11.8 and 6.7 mg/kg and protective index (PI = TD_50_/ED_50_) value of 4.6 and 8.1, respectively [12].

**Table 1:** Quinoline anticonvulsant compounds. 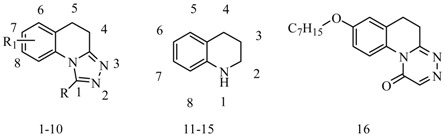

Compound No.	R	R_1_	Reference
**1**	-H	7-OCH_2_Ph(*4*-F)	[12]
**2**	-Ph	7-OCH_2_Ph	[13]
**3**	=O	7-OCH_2_Ph	[14]
**4**	=O	8-*n*-OC_6_H_13_	[15]
**5**	=O	8-*n*-OC_7_H_15_	
**6**	=O	7-*n*-OC_7_H_15_, 2-COC_2_H_5_	[16]
**7**	-H	5-Ph(3-F)	[17]
**8**	-H	7-*n*-C_6_H_13_, 5-Ph, 4=5	[18]
**9**	-H	5-*n*-OC_6_H_13_	[19]
**10**	-CONH_2_	7-*n*-OC_6_H_13_	[20]
**11**	5-(1,3,4-triazole), 8-*n*-OC_8_H_17_, 1=2 and 3=4		[21]
**12**	6-(1,3,4-triazole), 1-*n*-OC_6_H_13_, 2=O		[22]
**13**	1=2 and 3=4, 8-OCH_2_Ph, 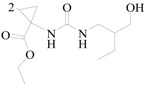		[23]
**14**	1=2 and 3=4, 8-OCH_2_Ph, 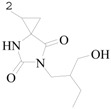		[24]
**15**	1=2 and 3=4, 2-Cl, 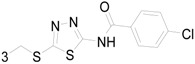		[25]

Cui *et al.*, reported a synthesis of 1-substituted-7-benzyloxy-4,5-dihydro-[1,2,4]triazolo[4,3-*a*]-quinolines. Anticonvulsant activity was evaluated in the MES test, *sc-*Met test, and rotarod neurotoxicity test. The safest compound was **2** (Table 1), with TD_50_ values of greater than 300 mg/kg which was better than most of the market drugs [13].

Jin *et al.*, prepared a novel type of 7-hydroxyl-3,4-dihydro-2(1*H*)-quinolines. In the anti-MES test, compound **3** showed ED_50_ of 12.3 mg/kg (Table 1), TD_50_ of 547.5 mg/kg, and the PI of 44.5 which was much greater than the PI of the reference drugs phenytoin, phenobarbital, carbamazepin and valproate [14]. Sun *et al.*, reported the synthesis of 8-alkoxy-4,5-dihydro-[1,2,4]triazole[4,3-*a*]quinoline-l-one derivatives and evaluated their anticonvulsant activities by MES test, *sc*-PTZ test, and rotarod test. The results demonstrated that compound **4** and compound **5** were the most potent anticonvulsants (Table 1), with ED_50_ values of 17.17 mg/kg and 24.55 mg/kg and PI values of 41.9 and 29.3 of compound **4** in the MES and *sc-*PTZ tests, respectively, and compound **5** having ED_50_ values of 19.7 mg/kg and 21.2 mg/kg and PI values of 36.5 and 33.9 in the MES and *sc*-PTZ tests, respectively. The PI values of compounds **4** and **5** were many folds better than that of the reference drugs which mentioned above, which have PI values in the range of 1.6–8.1 in the MES test and <0.22–5.2 in the *sc*-PTZ test [15].

Wei *et al.*, synthesized a series of 2-substituted-7-heptyloxy-4,5-dihydro-[1,2,4]triazolo[4,3-*a*]-quinolin-1(2*H*)-ones and evaluated their anticonvulsant activities. Pharmacological tests showed that compound **6** was the most active and also had the lowest toxicity (Table 1). In the anti-MES test, it showed ED_50_ of 8.2 mg/kg, TD_50_ of 318.3 mg/kg, and PI of 39.0 which was much greater than the PI of the reference drugs phenytoin and carbamazepine [16].

Guan *et al.*, designed and synthesized a new series of substituted quinoline-2(1*H*)-one and 1,2,4-triazolo[4,3-*a*]quinoline derivatives. Their anticonvulsant activities were evaluated by MES test, *sc*-PTZ test and rotarod test. Compound **7** showed the strongest anticonvulsant effect with ED_50_ of 27.4 mg/kg and 22.0 mg/kg in the anti-MES and anti-PTZ test, respectively (Table 1) [17].

Guan *et al.*, reported the synthesis of a series of novel 5-phenyl-[1,2,4]triazolo[4,3-*a*]quinoline derivatives and evaluated their anticonvulsant activities. The MES test showed that compound **8** was found to be the most potent compound with an ED_50_ value of 6.5 mg/kg and a PI value of 35.1 which was much higher than the PI of the reference drug phenytoin (Table 1) [18].

Guo *et al.*, synthesized a series of 5-alkoxy-[1,2,4]triazolo[4,3-*a*]quinoline derivatives. Their anticonvulsant activities were evaluated by MES test and their neurotoxicity was measured by the rotarod test. The results demonstrated that compound **9** was the most potent anticonvulsant (Table 1), with ED_50_ of 19.0 mg/kg and PI value of 5.8 in the MES test [19].

Sun *et al.*, synthesized a series of 8-alkoxy-5,6-dihydro-[1,2,4]triazino[4,3-*a*]quinolin-1-one derivatives and evaluated their activities. The results showed that compound **10** was the most potent with an ED_50_ value of 11.4 mg/kg (Table 1), TD_50_ of 114.1 mg/kg, PI value of 10.0 which is much greater than the PI of the reference drug carbamazepine [20].

Wei *et al.*, established a series of 1-formamidotriazolo[4,3-*a*]quinoline derivatives and evaluated their anticonvulsant activities. Compound **11** showed an ED_50_ of 30.1 mg/kg (Table 1), TD_50_ of 286 mg/kg, and PI of 9.5 which is greater than the reference drug carbamazepine with the PI value of 6.0 [21].

Wang *et al.*, synthesized two series of 8-alkoxy-5-(4*H*-1,2,4-triazol-4-yl)quinolines and 8-alkoxy-5-(2*H*-1,2,4-triazol-3-one-4-yl)quinolines. The anticonvulsant activities of these compounds were evaluated with MES test and rotarod test. Among the synthesized compounds, compound **12** was the most active, with and ED_50_ of 8.80 mg/kg (Table 1), TD_50_ of 176.03 mg/kg and PI value of 20.0. Its neurotoxicity was the lowest among the synthesized compounds. Meanwhile, it was also significantly lower than carbamazepine that used as reference. Beyond that, the broad spectrum activity of compound **12** was inferred from the anti-seizure results of bicuculline-, PTZ- and 3-mercaptopropionic acid-induced seizure tests [22].

Deng *et al.*, reported the synthesis of a series of 1-substituted-6-(4*H*-1,2,4-triazol-4-yl)-3,4-dihydroquinolin-2(1*H*)-ones and screened their anticonvulsant activities. In the MES screening, compound **13** showed anticonvulsant activity in moderation (Table 1). At the dose of 100 mg/kg, all the animals were protected from seizure after treatment with compound **13**, and all compounds synthesized exhibited no neurotoxicity [23].

He *et al.*, synthesized 16 new 1-(2-(8-(benzyloxy)quinolin-2-yl)-1-butyrylcyclopropyl)-3-substituted urea derivatives and tested their anticonvulsant activity using the MES test and *sc*-PTZ screening. The most active compound **14** showed anti-MES activity with an ED_50_ value of 14.3 mg/kg and TD_50_ value of 434 mg/kg after i.p. injection to mice (Table 1), which showed compound **14** with a PI of 30.3 in the MES test [24].

He *et al.*, prepared series of 16 new1-(8-(benzyloxy)quinolin-2-yl-6-substituted-4,6-dia-zaspiro [2,4]heptane-5,7-diones and evaluated their anticonvulsant activities using the MES and *sc*-PTZ tests. The most active compound **15** showed the MES-induced seizures with ED_50_ value of 8.6 mg/kg and TD_50_ value of 365.3 mg/kg after i.p. to mice (Table 1), compound **15** with a PI value of 26.8 in the MES test [25].

Kumar *et al.*, demonstrated synthesis of a series of quinoline-incorporated substituted thiadiazole and evaluated their anticonvulsant activity. Compound **16** showed protection against the MES model at 30 mg/kg and showed activity at both 0.5 and 4 h period at dose level of 30 mg/kg indicating the compound to be highly potent and long acting (Table 1) [26].

## 3. The Quinazoline or Quinazolinone Functional Groups

As new horizons in anticonvulsant therapy, the quinazolines and quinazolinone structural class has been proved to be useful for the design and development of potent anticonvulsant agents [27,28].

Wang *et al.*, synthesized several series of novel 5-alkoxytetrazolo[1,5-*a*]quinazoline derivatives. Anticonvulsant activities were evaluated using the MES test. Compound **17** protected completely against MES-induced seizure at a dose of 100 mg/kg (Table 2), and was the best active compound in this series [29].

Zheng *et al.*, prepared a series of novel 5-phenyl-[1,2,4]triazolo[4,3-*c*]quinazolin-3-amine derivatives and screened their anticonvulsant activities by the MES test and their neurotoxicity was evaluated by the rotarod neurotoxicity test. The most promising compound was **18** (Table 2), which showed an ED_50_ value of 27.4 mg/kg and a PI value of 5.8. These values were superior to those provided by valproate (ED_50_ and PI values of 272 and 1.6, respectively) in the MES test in mice [30].

El-Azab *et al.*, established a new series of 2,3,8-trisubstituted-4 (3*H*)-quinazoline derivatives. Compounds **19**, **20** and **21** displayed median LD_50_ values of 1000, 418 and 501 mg/kg with therapeutic index (LD_50_/ED_50_) values 10.2, 1.53 and 3.34 (Table 2). Compounds **19**, **20** and **21** showed better anticonvulsant activity and much lower toxicity comparable with the reference drugs valproate and methaqualone [31].

El-Azab *et al.*, reported a novel series of 7-substituted-4(3*H*)-quinazolinone and evaluated their antitumor and anticonvulsant activities. Compounds **22**, **23**, **24**, **25**, **26** and **27** showed advanced anticonvulsant activity as well as lower neurotoxicity than reference drugs valproate and methaqualone (Table 2) [32].

Abbas *et al.*, designed and synthesized a series of 2,3-disubstituted quinazolinone derivatives and a [1,2,4]triazino[2,3-*c*]quinazolinone and screened their anticonvulsant activity using the *sc*-PTZ and MES models. The study showed the most active compound **28** had a protective dose 50 (PD_50_) of 200.53 μmol/kg (PD_50_ of phenobarbitone = 62.18 μmol/kg) (Table 2) [33].

Rajasekaran *et al.*, synthesized a series of ten novel derivatives of 3-substituted-2-thioxoquinazolin-4(3*H*)-ones. The titled compounds were evaluated for anticonvulsant activities by MES test. The compounds **29** and **30** showed potent anticonvulsant activity (Table 2) [34].

Prashanth *et al.*, reported a novel class of *N*-substituted glycosmicine derivatives and evaluated their anticonvulsant activity by MES test and their neurotoxic effects were determined by rotorod test in mice. The most active compounds **31** and **32** exhibited anticonvulsant activity against MES-induced seizure at the dose of 100 mg/kg (Table 2). Among all compouds **31** and **32** were recorded 70% of protection [35].

Malik *et al.*, prepared various *N*-(benzo[d]thiazol-2-ylcarbamoyl)-2-methyl-4-oxoquinazoline-3(4*H*)-carbothioamide derivatives and evaluated their anticonvulsant activity with MES and *sc*-PTZ models in mice. The most active one was compound **33** with ED_50_ value of 82.5 mmol/kg (MES) and 510.5 mmol/kg (*sc-*PTZ) (Table 2). This molecule was more potent than phenytoin and ethosuximide which were used as reference antiepileptic drugs [36].

Saravanan *et al.*, demonstrated some novel quinazolinone derivatives and screened their antiepileptic activity using MES and *sc*-PTZ seizure tests. The most active one was compound **34** that revealed protection in MES at a dose of 30 mg/kg (ip) after 0.5 and 4 h (Table 2). This molecule also provided protection in the *sc*-PTZ at a dose of 100 mg/kg (0.5 h) and 300 mg/kg (4 h) [37].

**Table 2:** Anticonvulsant activity of quinazoline or quinazolinone compounds. 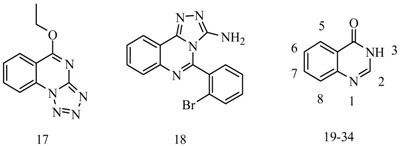

Compound No.	Substituent Group	Reference No.
**19**	2-CH_3_, 3-PH(2-CH_3_), 8-OCHCONHNH_2_	[31]
**20**	2-CH_3_, 3-PH(2-CH_3_), 8-OCHCONHNHCOOC_2_H_5_	
**21**	2-CH_3_, 3-PH(2-CH_3_), 8-OCHCONHNHCSSCH_3_	
**22**	2-CH_3_, 3-PH(2-CH_3_), 8-NHCOOC_2_H_5_	[32]
**23**	2-CH_3_, 3-PH(2-CH_3_), 8-NHCHPh(4-F)	
**24**	2-CH_3_, 3-PH(2-CH_3_), 8-NHCHPh(4-Cl)	
**25**	2-CH_3_, 3-PH(2-CH_3_), 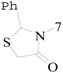	
**26**	2-CH_3_, 3-PH(2-CH_3_), 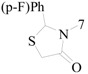	
**27**	2-CH_3_, 3-PH(2-CH_3_), 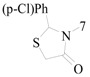	
**28**	2-CH_2_OPh(2,4-Cl_2_), 3-NHCOCH_2_NHNHCOC_5_H_4_N	[33]
**29**	3-Ph, 2-=S, 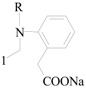 (R=1,3-dichlorobenzene)	[34]
**30**	3-naphthalene, 2-=S, 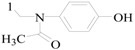	
**31**	1-CH_3_, 2-=O, 3-COC_2_H_4_NHPh(4-Cl)	[35]
**32**	1-CH_3_, 2-=O, 3-COC_2_H_4_NHPh(4-F)	
**33**	2-CH_3_, 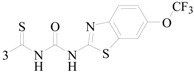	[36]
**34**	2-CH_3_, 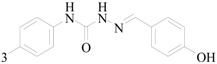	[37]

## 4. The Thiazole or Benzothiazole Functional Groups

In the past few decades, the literature has been enriched with progressive findings about the anticonvulsant activities of various substituted thiazole derivatives [38,39].

Siddiqui *et al.*, prepared a series of 1,3-benzothiazol-2-yl-semicarbazones and evaluated their anticonvulsant activity. Compounds **35**, **36** and **37** had shown 100% protection at both the time intervals, that is, 0.5 and 4 h (Table 3). None of the compounds had shown the sign of neurotoxicity [40].

**Table 3:** Anticonvulsant thiazole or benzothiazole compounds. 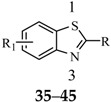

Compound No.	R	R_1_	Reference
**35**	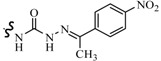	6-CH_3_	[40]
**36**		6-OCH_3_	
**37**	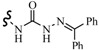		
**38**	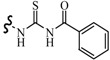	6-F	[41]
**39**	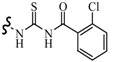	6-CH_3_	
**40**	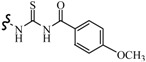	6-Br	
**41**	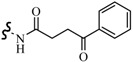	6-OCH_3_	[42]
**42**	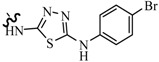	5,6-Cl2	[43]
**43**	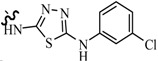		
**44**	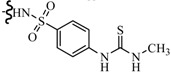	6-Cl	[44]
**45**	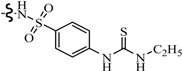		

Rana *et al.*, prepared a series of 1,3-benzothiazol-2-yl-benzamides and evaluated their anticonvulsant activity. Compounds **38**, **39**, **40** emerged as anticonvulsants with no neurotoxicity and can be claimed to detect compounds possessing effects against generalized toniceclonic (grand mal) and generalized absence (petit mal) seizures, respectively (Table 3) [41].

Hassan *et al.*, had reported synthesis of a series of *N*-(substituted benzothiazol-2-yl)amide derivatives and evaluated their anticonvulsant effect. Compound **41** emerged as the most effective, with median doses of 40.96 mg/kg (MES ED_50_), 85.16 mg/kg (*sc*-PTZ ED_50_) and 347.6 mg/kg (TD_50_) (Table 3) [42].

Siddiqui *et al.*, demonstrated a synthesis of various *N*-(5-chloro-6-substituted-benzothiazol-2-yl)-*N*′-(substituted phenyl)-[1,3,4]thiadiazole-2,5-diamines. All the newly synthesized compounds were screened for their anticonvulsant activity and were compared with the standard drug phenytoin sodium. Compounds **42** and **43** showed complete protection against MES-induced seizures (Table 3) [43].

Siddiqui *et al.*, also synthesized a series of sulphonamide derivatives and evaluated their possible anticonvulsant activity and neurotoxicity. Compounds **44** and **45** were active at lower doses of 100 and 30 mg/kg, respectively, after 4.0 h (Table 3). Compounds **44** and **45** showed activity at 300 mg/kg after 4 h in *sc*-PTZ screening. Two compounds **44** and **45** showed delayed toxicity that was toxic only after 4.0 h, which were comparable with that of Carbamazepine (300 mg/kg) [44].

**Figure 1:**
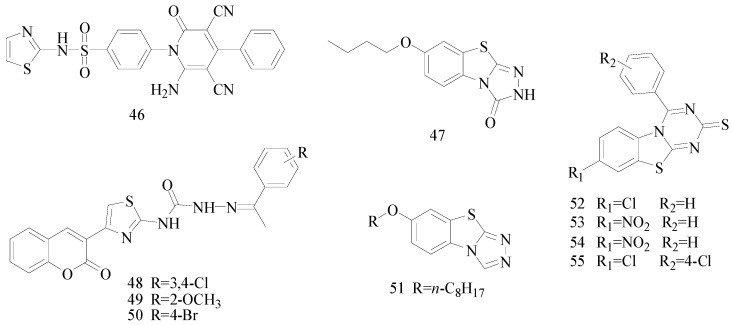
Structures of compounds **46**–**55**.

Farag *et al.*, reported many derivatives of heterocyclic compounds containing a sulfonamide thiazole moiety and evaluated the anticonvulsant effect. Compound **46** obviously showed anticonvulsant activity with no tonic stretching stage and protected all the animals tested (Figure 1) [45].

Siddiqui *et al.*, designed and synthesized several heteroaryl semicarbazones. All synthesized compounds were tested for anticonvulsant activity utilizing pentylenetetrazole-induced seizure (PTZ) and MES tests. Three compounds of the series, **47**, **48** and **49**, exhibited significant anticonvulsant activity at dose of 30 mg/kg comparable to the standard drug phenytoin (Figure 1) [46].

Liu *et al.*, established a new series of 7-alkoxy[1,2,4]triazolo[3,4-*b*]benzothiazol-3(2*H*)-ones and evaluated their anticonvulsant activities. Compound **50** was the most active in MES-induced seizure test with ED_50_ value of 13.6 mg/kg (Figure 1). Meanwhile, its neurotoxicity was extremely low, with PI > 51 [47].

Deng *et al.*, reported synthesis of 7-alkoxy-triazolo-[3,4-*b*]benzo[*d*]thiazoles. In the MES test, most of the compounds synthesized showed good effects on convulsion. Among the compounds studied, compound **51** was found to be the most potent compound with ED_50_ value of 8.0 mg/kg and PI value of 15.0 (Figure 1), possessing better anticonvulsant activity and higher safety than market drugs carbamazepine and phenytoin. Compound **51** exhibited activities of broad spectrum in several animal models [48].

Siddiqui *et al.*, synthesized a number of new 8-substituted-4-(2/4-substituted phenyl)-2*H*-[1,3,5]triazino[2,1-*b*][1,3]benzothiazole-2-thiones and evaluated their anticonvulsant in a mouse seizure model and were comparable with the standard drug phenytoin. Compounds **52**, **53**, **54** and **55** showed complete protection after time periods of 0.5 h and 4 h (Figure 1) [49].

## 5. The Benzothiazines or Benzoxazinone Functional Groups

**Figure 2:**
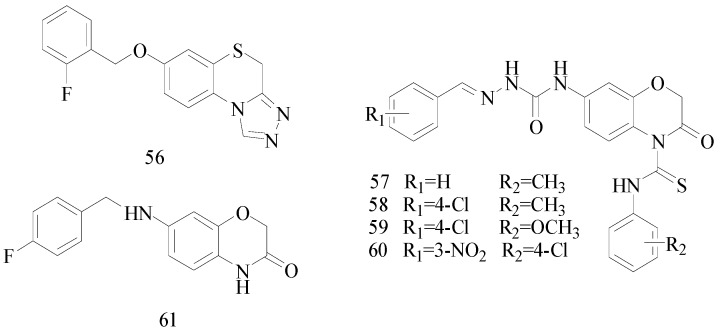
Structures of compounds **56**–**61**.

Zhang *et al.*, synthesized a novel series of 7-alkoxy-2*H*-1,4-benzothiazin-3(4*H*)-ones and a new series of 7-alkoxy-4*H*-[1,2,4]triazolo[4,3-*d*]benzo[*b*][1,4]thiazine derivatives. The anticonvulsant activity of these compounds was evaluated by MES test and tarod test following intraperitoneal injection in KunMing mice. Compound **56** was the most active compound, with an ED_50_ of 17.0 mg/kg, TD_50_ of 243.9 mg/kg and PI of 14.3 (Figure 2). The neurotoxicity was the lowest among the synthesized compounds. Meanwhile, it was also significantly lower than carbamazepine that was used as reference. [50].

Siddiqui *et al.*, reported a series of (*Z*)-2-(substituted aryl)-*N*-(3-oxo-4-(substituted carbamothioyl)-3,4-dihydro-2*H*-benzo[*b*][1,4]oxazin-7-yl)hydrazine carboxamides. The anti-convulsant activity was assessed by the MES test, *sc*-PTZ test and intraperitoneal thiosemicarbazide test (i.p. TSC). Compounds **57**, **58**, **59** and **60** were the most active ones, protecting 83%–100% of the animals against MES-induced seizures (Figure 2), and also exhibited potent anticonvulsant activity in chemical-induced seizures [51].

Piao *et al.*, prepared a series of 7-benzylamino-2*H*-1,4-benzoxazin-3(4*H*)-ones. Their anticonvulsant activities were evaluated by the MES test and their neurotoxicity was evaluated by the rotarod neurotoxicity test. The MES test showed that compound **61** was the most potent with ED_50_ value of 31.7 mg/kg and PI value of 7.2 (Figure 2) [52].

## 6. The Oxadiazole or Benzoxazinone Functional Groups

The oxadiazole scaffold is very versatile and has been subjected to extensive study in recent years. Compounds containing oxadiazole rings have been studied for many biological activities [53].

Bhat *et al.*, prepared a series of 3-(4-acetyl-5*H*/methyl-5-substituted phenyl-4,5-dihydro-1,3,4-oxadiazol-2-yl)-2*H*-chromene-2-ones and evaluated their anticonvulsant activity and neurotoxicity. Compound **62** was found to be potent and had activity at a lower dose of 30 mg/kg in MES-test (Table 4). All the compounds were less toxic as compared with the standard drug phenytoin [54].

Tabatabai *et al.*, synthesized a series of some derivatives of 2-(2-phenoxy)phenyl-1,3,4-oxadiazole. Although the most effective compound **63** was a weaker anticonvulsant than diazepam (Table 4), it should be mentioned that it had a good margin of safety and LD_50_, which were 15-fold its ED_50_ [55].

Harish *et al.*, reported a series of novel 1-[5-(4-methoxyphenyl)-[1,3,4]oxadiazol-2-yl]-piperazine derivatives. The anticonvulsant effects of these derivatives on MES-induced seizures were experimented in male Wistar rats and phenytoin was used as reference drug. Compounds **64**, **65**, **66** and **67** showed excellent anticonvulsant activity in MES model (Table 4) [56].

**Table 4:** Anticonvulsant oxadiazole or benzoxazinone compounds. 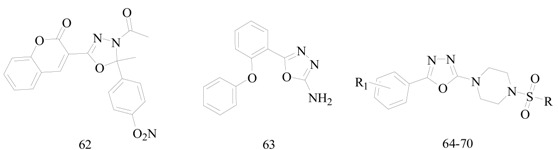

Compound No.	R	R_1_	Reference
**64**	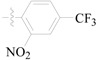	4-OCH_3_	[56]
**65**	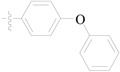	4-OCH_3_	
**66**	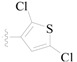	4-OCH_3_	
**67**	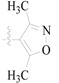	4-OCH_3_	
**68**	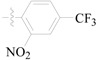	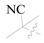	[57]
**69**	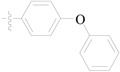	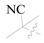	
**70**	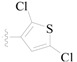	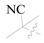	

Harish *et al.*, investigated a series of new 2-methyl-2-[3-(5-piperazin-1-yl-[1,3,4]oxadiazol-2-yl)-phenyl]-propionitrile derivatives. All the compounds were screened for their anticonvulsant activity against MES seizure and their neurotoxic effects were determined by rotorod test. Compounds **68**, **69** and **70** were found to be the most potent of this series (Table 4). These compounds showed no neurotoxicity at the maximum dose administered (100 mg/kg) [57].

Siddiqui *et al.*, reported a synthesis of new 5-(1*H*-indol-3-yl)methyl-4-(substituted-aryl)-2,4-dihydro-3*H*-1,2,4-triazole-3-thiones. All the newly synthesized compounds were screened for their anticonvulsant activity in the MES model and were compared with the standard drugs phenytoin sodium and carbamazepine. Among these compounds, **71** was found to be the most active in the series that showed protection against seizures both after 0.5 h and 4 h at 30 mg/kg body mass (Table 4) [58].

**Figure 3:**
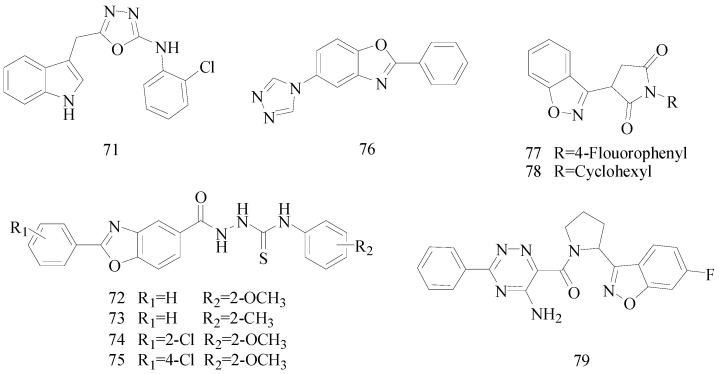
Structures of compounds **71**–**79**.

Siddiqui *et al.*, designed and synthesized a series of 5-carbomethoxybenzoxazole derivatives. Compounds **72**, **73**, **74** and **75** were found to be more lipophilic (Figure 3), thus having potent anticonvulsant activity [59].

Wei *et al.*, demonstrated a synthesis of novel 2-substituted-6-(4*H*-1,2,4-triazol-4-yl)benzo[*d*] oxazoles and evaluated the anticonvulsant activity with the MES test and *sc*-PTZ test. Compound **76** was the most active and also had the lowest toxicity (Figure 3). In the anti-MES potency test, it showed ED_50_ of 29.5 mg/kg, a TD_50_ of 285 mg/kg, and a PI of 9.7 which was greater than the reference drug, carbamazepine that has a PI of 6.4 [60].

Malik *et al.*, prepared a series of 3-(benzo[*d*]isoxazol-3-yl)-*N*-substituted pyrrolidine-2,5-dione and evaluated their anticonvulsant activities. Preliminary anticonvulsant activity was performed using MES and *sc*-PTZ tests after ip injection into mice. ED_50_ value of compound **77** was 14.90 mg/kg (Figure 3). Similarly the most potent one in *sc*-PTZ was compound **78** with an ED_50_ value of 42.30 mg/kg (Figure 3). These compounds were more active and had lower neurotoxicity than the control drugs ethosuximide and phenytoin [61].

Malik *et al.*, synthesized a novel series of (5-amino-3-substituted-1,2,4-triazin-6-yl)(2-(6-halo-substituted-benzo[d]isoxazol-3-yl) pyrrolidin-1-yl)methanone. The MES test showed that compound **79** was the most potent compound (Figure 3), with an ED_50_ value of 6.20 mg/kg (oral/rat) and a PI value of >48.38 which was much higher than the PI of the reference drug phenytoin [62].

## 7. The Pyridine Functional Group

**Figure 4:**
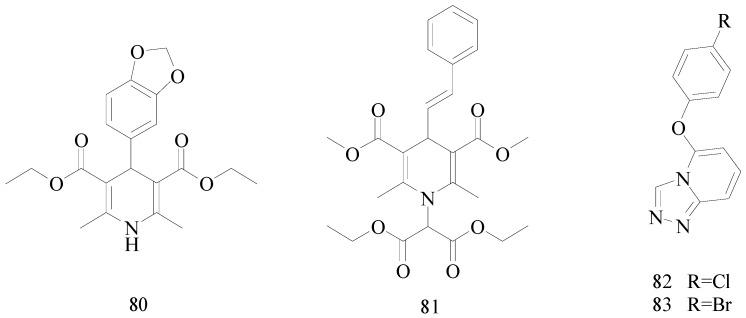
Structures of compounds **80**–**83**.

Pyridines and substituted pyridines are an important family of heterocyclic compounds that has attracted significant interest in medicinal chemistry in recent years [63]. Prasanthi *et al.*, reported synthesis of dialkyl 4-(benzo[*d*][1,3]dioxol-6-yl)-1,4-dihydro-2,6-dimethyl-1-substituted pyridine-3,5-dicarboxylates. The present study revealed that compound **80** showed promising anticonvulsant activity compared to phenytoin (Figure 4). Further, the prediction data of the molecular properties supports that compound **80** might involve hydrogen bonding interaction with target site, and displayed good binding in silico absorption and lower binding rate of plasma to protein, which made it to be a good candidate for treatment of epilepsy [64].

Prasanthi *et al.*, prepared a series of new *N*-diethylmalonyl derivatives of nifedipine and other isosteric analogues. Anticonvulsant screening was performed using *sc*-PTZ and MES induced seizures. The majority of the compounds were effective in *sc*-PTZ and MES screening. Compound **81** showed good activity displaying the maximum protection (Figure 4) [65].

Guan LP *et al.*, described a series of new 5-alkoxy-[1,2,4]triazolo[4,3-*a*]pyridine derivatives and evaluated their anticonvulsant activity and neurotoxicity with the MES and rotarod tests, respectively. The most promising compounds, **82** and **83** showed ED_50_ values of 13.2 and 15.8 mg/kg and had a PI value of 4.8 and 6.9, respectively (Figure 4) [66].

## 8. The Pyrazole Functional Group

Pyrazole and its derivatives consist of five-membered heterocycles with two *ortho*-nitrogen-atoms. These compounds have received attention in the field of current medicinal and pharmacological research, and are reported to have a broad spectrum of biological activities, such as antitumor, antimicrobial, antioxidant and antimalarial activities [67,68,69,70].

**Figure 5:**
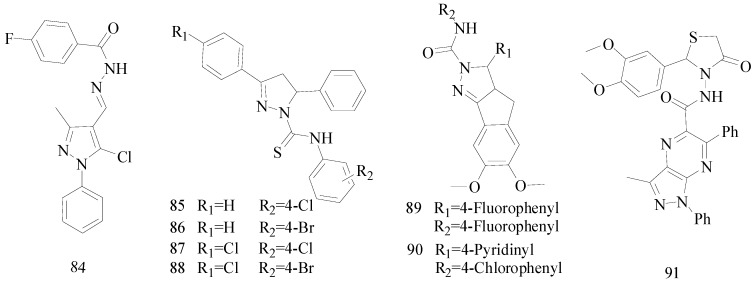
Structures of compounds **84**–**91**.

Kaushik *et al.*, established synthesis of *N*′-[(5-chloro-3-methyl-1-phenyl-1*H*-pyrazol-4-yl)methylene]2/4-substituted hydrazides and evaluated their anticonvulsant activity against MES- and *sc*-PTZ-induced seizure models in mice. All compounds showed protection in the MES model at 100 mg/kg, including compound **84** which showed activity at 0.5 h and 4.0 h periods indicating that **84** was potent having a rapid onset and long duration of action (Figure 5). Compound **84** showed activity at a dose of 100 mg/kg comparable to sodium valproate in the *sc*-PTZ test [71].

Siddiqui *et al.*, had reported various 3,5-(substituted-diphenyl)-4,5-dihydro-pyrazole-1-acid phenylamides and evaluated their anticonvulsant activities. Compounds **85**, **86**, **87** and **88** were found to protect 100% of the animals in the MES screening at a dose of 25 mg/kg (Figure 5). They were also found to have appreciable anticonvulsant activity in *sc*-PTZ screening [72].

Ahsan MJ *et al.*, designed and synthesized a series of fourteen 3a,4-dihydro-3*H*-indeno[1,2-*c*] pyrazole-2-carboxamide/carbothioamide analogues. Among the compounds synthesized, some exhibited marked effect on seizure model under minimal clonicity (6 Hz psychomotor seizure test). Compound **89** was found to be the most active compound of the series showing 75% (3/4, 0.25–2.0 h) and 50% (2/4, 4.0 h) protection against minimal clonic seizure at 100 mg/kg without any toxicity (Figure 5). Compound **90** showed protection in MES seizure and subcutaneous metrazol (*sc-*MET) seizure at 300 mg/kg (Figure 5) [73].

Farghaly A *et al.*, synthesized a series of new pyrazolo[3,4-*b*]pyrazines containing, 1,2,4-oxadiazolyl, thiadiazolyl, imidazothiadiazolyl, thiazolidinonyl, substituents and other different substituents. Compound **91** showed best results at reducing PTZ-induced tonic convulsions and mortality (Figure 5) [74].

## 9. The Imidazole Functional Group

Imidazole and its derivatives are a class of 5-membered heterocyclic structures having two non-adjacent nitrogen atoms. Recent studies revealed that the substituted imidazole derivatives have attracted much attention due to their broad spectrum of pharmacological activities such as anti-inflammatory, analgesic [75,76]. Literature survey shows that imidazole-heterocyclic compounds could be new classes of anticonvulsant agents by the virtue of their potential anticonvulsant properties [77].

**Figure 6:**
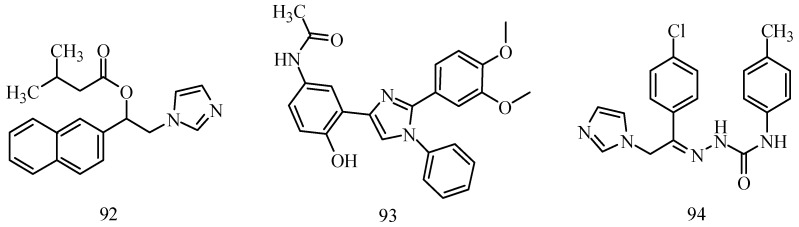
Structures of compounds **92**–**94**.

Karakurt *et al.*, described a series of 2-acetylnaphthalene derivatives. Quantification of anti-convulsant protection was calculated via the i.p. route (ED_50_ and TD_50_) for the most active candidate (**92**) (Figure 6). Observed protection in the MES model was 38.46 mg/kg and 123.83 mg/kg in mice and 20.44 mg/kg, 56.36 mg/kg in rats, respectively [78].

Husain *et al.*, established a synthesis of a series of 1,2,4-trisubstituted-1*H*-imidazole derivatives. Anticonvulsant activity was shown by the majority of the synthesized compounds in the MES and *sc*-PTZ screening when given i.p. to mice. In anticonvulsant screening, only one compound, **93**, showed potent activity comparable to that of standard drugs phenytoin and carbamazepine (Figure 6) [79].

Amir *et al.*, demonstrated synthesis of a series of novel imidazole incorporated semicarbazones. Compound **94** showed the highest activity among the compounds synthesized with no neurotoxic and depressant effects on CNS (Figure 6). Liver enzyme estimations (serum glutamate oxaloacetate transaminase (SGOT), serum glutamate pyruvate transaminase (SGPT), alkaline phosphatase) of the compound also showed no significant change in the enzyme levels [80].

Ulloora *et al.*, prepared a variety of five new series of imidazo[1,2-*a*]pyridines carrying biologically active pyrazoline, cyanopyridone, cyanopyridine, 2-aminopyrimidine and pyrimidine-2-thione systems. The target compounds were screened for their *in vivo* anticonvulsant activity following MES and *sc*-PTZ methods at a small test dose of 10 mg/kg. Compounds **95**, **96**, **97**, **98**, **99** and **100** displayed potent anticonvulsant activity without displaying any toxicity (Table 5) [81].

Ulloora *et al.*, designed and synthesized new 2-arylimidazo[1,2-*a*]pyridines carrying suitably substituted 1,2,3-triazoles. The anticonvulsant study was carried out by MES and *sc*-PTZ screening methods, while their toxicity study was performed following rotarod method. The most active was compound **101** which displayed reasonably good activity in both the durations of 0.5 and 4 h indicating that they possess rapid onset and long duration of action (Table 5). It exhibited complete protection against seizure and their activity at 20 mg/kg was comparable with that of standard drug diazepam [82].

**Table 5:** Anticonvulsant imidazole compounds. 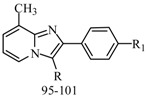

Compound No.	R	R_1_	Reference
**95**	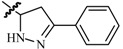	-F	[81]
**96**	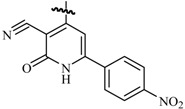		
**97**	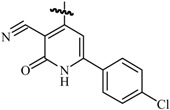		
**98**	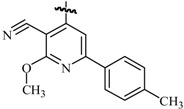		
**99**	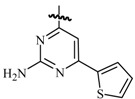	-H	
**100**	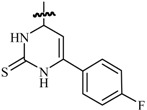	-H	
**101**	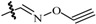	-CH_3_	[82]

## 10. The Pyrimidine Functional Group

Pyrimidine is an aromatic heterocyclic organic compound similar to pyridine. One of the three diazines, six-membered heterocyclics with two nitrogen atoms in the ring, has the nitrogens at positions 1 and 3 in the ring. Pyrimidines that have a broad spectrum of bioactivities (antibacteria, anticancer and anti-inflammation and so on) are an important one of the heterocyclic compounds [83,84,85].

**Figure 7:**
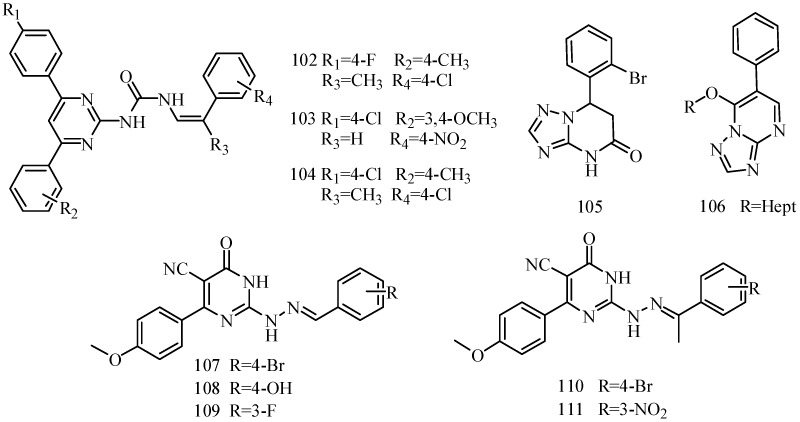
Structures of compounds **102**–**111**.

Alam *et al.*, synthesized a number of *N*-(4,6-substituted diphenylpyrimidin-2-yl) semicarbazones and tested their anticonvulsant activity against the two seizure models, MES and *sc*-PTZ. Three compounds (**102**, **103** and **104**) were found to be significantly active as they showed protection at the lowest dose of 30 mg/kg after 0.5 h and did not show any sign of neurotoxicity except in case of compound **102** which was found to be neurotoxic at 300 mg/kg after 4.0 h (Figure 7) [86].

Deng *et al.*, described the synthesis and anticonvulsant activities of 7-(substituted-phenyl)-6,7-dihydro-[1,2,4]triazolo[1,5-*a*]pyrimidin-5(4*H*)-ones and their derivatives. The majority of the compounds synthesized showed inhibition effects on MES-induced convulsion. The most promising compound **105** showed significant anticonvulsant activity in MES test with ED_50_ value of 19.7 mg/kg (Figure 7). It was safer than reference drugs with much higher PI value. In addition, the protective effect of compound **105** against seizures induced by PTZ, ISO, TSC, 3-MP, and bicuculline in the chemical-induced seizure tests suggested that compound **103** displayed broad spectrum activity in several models [87].

Jiang *et al.*, reported a novel series of 7-substituted-5-phenyl-[1,2,4]triazolo[1,5-*a*] pyrimidines. Their anticonvulsant activities were measured through the MES test, and carbamazepine (ED_50_ = 11.8 mg/kg) and valproate (ED_50_ = 272 mg/kg) were used as the reference drugs. Amongst the compounds tested, compound **106** was the most active in inhibiting convulsion with ED_50_ value of 84.9 mg/kg that was higher than valproate but lower than carbamazepine (Figure 7) [88].

Shaquiquzzaman *et al.*, established syntheses of some new pyrimidine-5-carbonitrile derivatives. In the MES test, compounds **107**, **108** and **109** were found to be highly active at a dose level of 30 mg/kg at 0.5 h time interval (Figure 7), indicating their ability to prevent seizure spread at a relatively low dose [89]. Shaquiquzzaman *et al.*, also reported a series of dihydropyrimidine-5-carbonitrile derivatives and evaluated their anticonvulsant activity against MES and *sc*-PTZ models. Compounds **110** and **111** were found to be most active showing activity both in MES and *sc*-PTZ screen at lower doses of 30 mg/kg at 0.5 h and 100 mg/kg at 4 h (Figure 7). In the rotarod motor impairment screen, compound **110** did not show any motor impairment, even at the maximum dose of 300 mg/Kg. The pharmacophore hypothesis also fits best for compounds **110** and **111** [90].

## 11. The Phthalazine Functional Group

As a heterocyclic compound, the molecular formula of phthalazine is C_8_H_6_N_2_. Because of the broad spectrum of bioactivities such as anticonvulsion, vasorelaxation, anti-inflammation and cardiotonic effect, its derivatives are generally used for treating disease [91,92,93].

**Figure 8:**
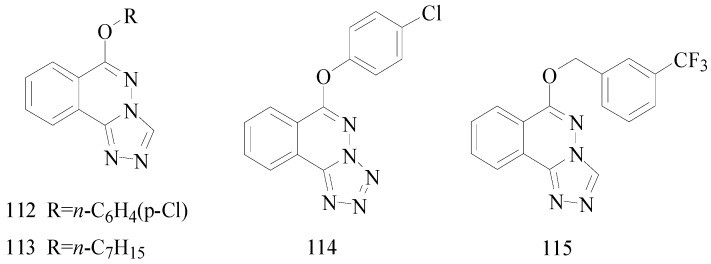
Structures of compounds **112**–**115**.

Zhang *et al.*, designed and synthesized a new series of 6-alkoxy-[1,2,4]triazolo[3,4-*a*] phthalazines and evaluated their anticonvulsant activity and neurotoxicity by the MES test and the rotarod test respectively. The most promising compounds **112** and **113** showed a median effective dose of 7.1 and 11.0 mg/kg (Figure 8), and had protective index values of 5.2 and 8.0, respectively. The two compounds were further found to have potent activity against seizures induced by PTZ, ISO, TSC, 3-MP but not seizures induced by strychnine [94].

Sun *et al.*, investigated a new phthalazine tetrazole derivative. Compound **114** exhibited higher activity (ED_50_ = 6.8 mg/kg) and lower neurotoxicity (TD_50_ = 456.4 mg/kg) (Figure 8), resulting in a higher PI = 67.1 compared with carbamazepine (PI = 6.4). In addition, compound **114** exhibited significant oral anticonvulsant activity (ED_50_ = 24 mg/kg) against MES-induced seizure with low neurotoxicity (TD_50_ > 4500 mg/kg) in mice, resulting in a PI value of more than 187.5. Compound **114** was also tested in chemically induced animal models of seizure (PTZ, ISO, TSC and 3-MP) to further investigate the anticonvulsant activity. Compound **114** produced significant anticonvulsant activity against seizures induced by ISO, TSC and 3-MP [95].

Bian *et al.*, reported a synthesis of new 6-substituted-[1,2,4]triazolo[3,4-*a*](tetrazolo[5,1-*a*]) phthalazine derivatives. All the compounds were evaluated for their anticonvulsant activities using the MES test. The most promising compound **115** showed significant anticonvulsant activity in MES test with ED_50_ value of 9.3 mg/kg (Figure 8). It displayed a wide margin of safety with protective index much higher than the standard drug carbamazepine [96].

## 12. The Triazine Functional Group

Triazine is a six membered heterocyclic ring compound containing three nitrogen atoms. The triazine moiety has also attracted the attention of chemists because many triazines are biologically active and are used in medicine, especially as anti-AIDS, anticancer, and antitubercular agents, for their anti-anxiety and anti-inflammatory activities, as well as used in agriculture [97,98,99].

**Figure 9:**
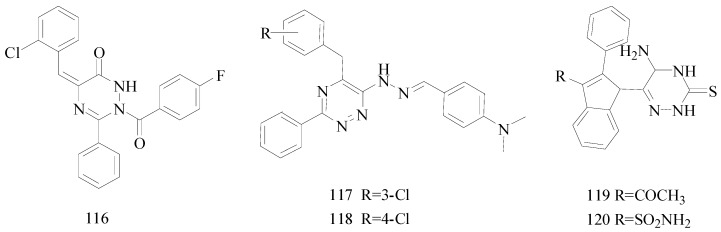
Structures of compounds **116**–**120**.

Kaushik *et al.*, designed and synthesized a new series of 2-(substituted aryloxy)-5-(substituted benzylidene)-3-phenyl-2,5-dihydro-1*H*-[1,2,4]triazin-6-one. Their anticonvulsant activity was evaluated by MES test, *sc*-PTZ test. Among the compound tested, compound **116** showed protection from seizures in both the animal models at dose level of 30 mg/kg (Figure 9). The compound **116** showed activity both at 0.5 h and 4 h periods at dose level of 30 mg/kg, indicating the compound to be highly potent and long acting [100].

Amir *et al.*, demonstrated synthesis of new hydrazone incorporated triazines and evaluated for their anticonvulsant activity through MES and *sc*-PTZ screenings. Among the tested compounds, compound **117** (MES ED_50_ 54.31, *sc*-PTZ ED_50_ 92.01) and compound **118** (MES ED_50_ 46.05, *sc*-PTZ ED_50_ 83.90) emerged as the most active anticonvulsant agent having GABA-ergic effects (Figure 9). Compounds **117** and **118** also showed less CNS depressant effect than the standard drug carbamazepine [101].

Ahuja *et al.*, synthesized a series of thirty indole C-3 substituted 5-amino-6-(5-substituted-2-phenyl-1*H*-indol-1-yl)-4,5-dihydro-1,2,4-triazine-3(2*H*)-thiones. Compound **119** had significant activity in the MES test with minimal duration of limb extension (5.40 ± 0.61 s) and quantitative median dose of 7 mg/kg. In *sc*-PTZ screening, compound **120** increased the seizure latency to clonic convulsion and with effective at a median dose of 35 mg/kg (Figure 9) [102].

## 13. The Triazolethione Functional Group

Many compounds bearing a triazole moiety were found to possess anticonvulsant properties in various animal models of epilepsy. Therefore, some people want to loop through a combination of triazole-thione compounds to improve the antiepileptic activity.

Luszczki *et al.*, reported the effects of 4-(4-bromophenyl)-5-(3-chlorophenyl)-2,4-dihydro-3*H*-1,2,4-triazole-3-thione (compound **121**) and 5-(3-chlorophenyl)-4-(4-methylphenyl)-2,4-dihydro-3*H*-1,2,4-triazole-3-thione (compound **122**) on the protective action of four classical antiepileptic drugs—carbamazepine, phenobarbital, phenytoin and valproate—against MES test in mice (Table 6). Results indicated that compound **121** administered intraperitoneally at doses of 75 and 100 mg/kg significantly elevated the threshold for electroconvulsions in mice. Compound **121** (50 mg/kg) significantly enhanced the anticonvulsant activity of carbamazepine, phenobarbital and valproate. Compound **122** administered intraperitoneally at 10 mg/kg significantly elevated the threshold for electroconvulsions in mice. Moreover, compound **122** (5 mg/kg) significantly enhanced the anticonvulsant activity of valproate, but not that of carbamazepine, phenobarbital or phenytoin in the MES test in mice. Pharmacokinetic experiments revealed that compound **122** significantly elevated total brain concentrations of valproate in mice [103,104].

**Table 6:** Structures of compounds **121**–**126**. 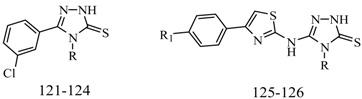

Compound No.	R	R_1_	Reference
**121**	-Ph(*p*-Br)	—	[103,104]
**122**	-Ph(*p*-CH_3_)	—	
**123**	*n*-C_6_H_13_	—	[105]
**124**	-Ph(*p*-F)	—	
**125**	-Ph(*o*-CH_3_)	-Cl	[106]
**126**	-Ph(*p*-OCH_3_)	-Br	[107]

Siddiqui *et al.*, prepared a various of 3-[4-(substituted phenyl)-1,3-thiazol-2-ylamino]-4-(substituted phenyl)-4,5-dihydro-1*H*-1,2,4-triazole-5-thiones. Their *in vivo* anticonvulsant screenings were performed using the two most adopted seizure models, MES and *sc*-PTZ. Two compounds, **123** and **124** (Table 6), showed significant anticonvulsant activity in both the screenings with ED_50_ values of 23.9 mg/kg and 13.4 mg/kg, respectively, in the MES screen and 178.6 mg/kg and 81.6 mg/kg, respectively, in the *sc*-PTZ test. They displayed a wide margin of safety with PI, median hypnotic dose (HD_50_) and median lethal dose (LD_50_) which were much higher than that of the standard drugs [105].

Plech *et al.*, designed and synthesized 4-alkyl-1,2,4-triazole-3-thione derivatives. A group of derivatives showed strong anticonvulsant activity. The characteristic features of the most active compounds were rapid onset and long lasting effects. Among the tested compounds, compound **125** was assayed for the different PI values at different preprocessing times (Table 6), and the results of that were ranging from 2.8 to 9.7 [106].

Plech *et al.*, also reported a synthesis of 1,2,4-triazole-3-thione derivatives. Characteristic features of all active compounds were a rapid onset of action and long lasting effects. Compound **126** exhibited the most potent activity (ED_50_ = 35.2 mg/kg) (Table 6) [107].

## 14. The Indoline-2,3-dione Functional Group

Isatin (indoline-2,3-dione), one of the simplest indole derivatives, has led to numerous analogues with a wide range of biological properties, including anxiogenic, sedative, anticonvulsant, anticancer activities [108,109].

**Figure 10:**
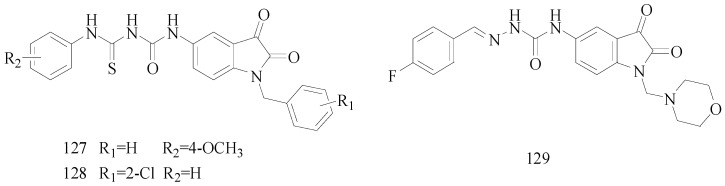
Structures of compounds **127**–**129**.

Siddiqui *et al.*, designed various 1-(amino-*N*-arylmethanethio)-3-(1-substituted-benzyl-2,3-dioxoindolin-5-yl) ureas. Their *in vivo* anticonvulsant screenings were performed by the two most adopted seizure models, MES and *sc*-PTZ. At 300 mg/kg, compounds **127** and **128** showed significant protective effect on MES- and *sc*-PTZ-induced seizures (Figure 10). Even at the lower dose of 100 mg/kg, compound **128** exhibited good protection on MES-induced seizure. These two compounds exhibited marked protective effect against seizures in a 6 Hz psychomotor seizure test, and could be used as lead compounds for future investigations [110].

Prakash *et al.*, prepared a series of 1-(substituted benzylidene)-4-(1-(morpholino/piperidino methyl)-2,3-dioxoindolin-5-yl) semicarbazides. The compounds were subjected to *in vivo* antiepileptic evaluation using MES and *sc*-PTZ test methods. The neurotoxicity was determined by rotarod test. Among the synthesized compounds, **129** revealed excellent protection in both models with lower neurotoxicity (Figure 10) [111].

## 15. The Cyclopropanecarboxylate Functional Group

**Figure 11:**
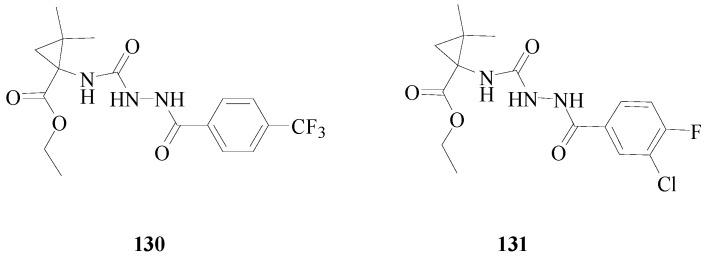
Structures of compounds **130**–**131**.

He *et al.*, synthesized twenty three 1-(2-arylhydrazinecarboxamido)-2,2-dimethylcyclopropane-carboxylate derivatives and tested their anticonvulsant activity using the MES, *sc*-PTZ screens. Their neurotoxicity was determined by applying the rotorod test. The most active compound **130** showed protection against the MES-induced seizures with ED_50_ value of 9.8 mg/kg and TD_50_ value of 332.2 mg/kg after i.p. to mice (Figure 11), which provided compound **128** with a PI of 33.9 in the MES test [112].

Zhong *et al.*, reported fourteen ethyl 2,2-dimethyl-1-(2-substituted-hydrazinecarboxamido) cyclopropanecarboxylate derivatives and tested the anticonvulsant activity using the MES, *sc*-PTZ screens. The most active compound **131** showed protection against MES-induced seizures with an ED_50_ value of 9.2 mg/kg and TD_50_ value of 387.5 mg/kg after i.p. to mice (Figure 11), which provided compound **129** with a PI of 42.1 in the MES test [113].

## 16. The Pyrrolidine-2,5-dione Functional Group

Derivatives of pyrrolidine-2,5-diones, as heterocyclic compounds, have been widely applied in medicinal chemistry and synthesis fields. They exhibit numerous bioactivities, especially in anticonvulsant and tyrosinase inhibitory activities. Therefore, development of new and efficient strategies for the synthesis of multi-substituted pyrrolidine-2,5-diones is also the current hot in organic and medical chemistry [114].

Obniska *et al.*, designed and synthesized many series pyrrolidine-2,5-diones (Table 7) and tested their anticonvulsant activity in the MES and metrazole seizure threshold (*sc*-Met) tests [115,116,117,118,119,120,121,122,123,124,125,126,127]. The quantitative evaluation in the MES seizures after oral administration into rats showed that the most active were compound **153** with ED_50_ of 7.4 mg/kg and compound **154** with ED_50_ of 26.4 mg/kg. These molecules were more potent and also less neuron-toxicity than that of phenytoin which was used as reference antiepileptic drug. Although Kaminski *et al.*, had reported several series pyrrolidine-2,5-diones (Table 7) used for anticonvulsant activity, none exhibited better than compound **153** [128,129,130,131,132].

**Table 7:** Structures of compounds **132**–**165**. 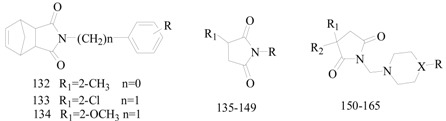

Comp. No.	R	R_1_	R_2_	X	Reference
**135**	-NHPh(*p*-CH_3_)	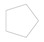	—	—	[116,117,118]
**136**	-NHPh(*o*-CF_3_)				
**137**	-NHPh				
**138**	-NHPh(2,4-Cl_2_)				
**139**	-NHPh(*m*-CH_3_)	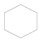			[116]
**140**	-NHPh(2,4-Cl_2_)				[117]
**141**	-Ph(*o*-OCH_3_)				[119]
**142**	-NHPh(4-Cl)				
**143**	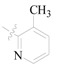	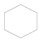			[120]
**144**		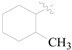			
**145**	-NHPh	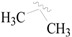			[121]
**146**	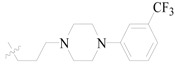	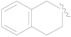			[122]
**147**		-Ph(*o*-CF_3_)			[123]
**148**	-N(C_2_H_4_)_2_N-CH_3_	-Ph(*m*-Cl)			[124]
**149**	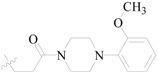	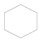			[125]
**150**	-Ph(*m*-CF_3_)	-H	-Ph(*m*-CH_3_)	N	[126,127,128]
**151**	-Ph(*p*-Cl)	-H	-Ph(*m*-Cl)		
**152**	-Ph(*m*-CF_3_)	-H	-Ph(*m*-Cl)		
**153**	-Ph(*p*-F)	-H	-Ph(*o*-Br)		
**154**	-Ph(*m*-Cl)	-H	-Ph(*o*-Br)		
**155**	-Ph	-H	-Ph(*m*-CF_3_)		[123,124,125,126,127,128]
**156**	-CH_2_Ph	-H	-Ph(*o*-Cl)	C	
**157**	—	-H	-Ph	O	[129]
**158**	-CH_2_CH_2_OH	-CH_3_		N	[130]
**159**	-CH_2_Ph			C	
**160**	-Ph(*p*-F)			N	[131]
**161**	-Ph(*m*-Cl)				
**162**	-Ph(*3*,*4*-Cl_2_)				
**163**	-Ph(*m*-CF_3_)	-Ph	-Ph		[132]
**164**	-Ph(*3*,*4*-Cl_2_)	-H	-CH_3_		[133]
**165**	-Ph(*3*,*4*-Cl_2_)	-H	-H		

Rybka *et al.*, reported a synthesis of 22 new *N*-[(4-phenylpiperazin-1-yl)-methyl]-3-methyl-pyrrolidine-2,5-dione and pyrrolidine-2,5-dione derivatives. Administration to mice revealed that the most active compounds were compound **164** with ED_50_ = 16.13 mg/kg (MES), ED_50_ = 133.99 mg/kg (*sc*-PTZ) and compound **165** with ED_50_ = 37.79 mg/kg (MES), ED_50_ = 128.82 mg/kg (*sc*-PTZ) (Table 7). Compared with the positive control drugs valproate and ethosuximide, these compounds exhibited more activity and less neurotoxicity [133].

## 17. The Imidazoline-2,4-dione Functional Group

Imidazoline-2,4-diones, also called hydantoins, a class of cyclic imides, have been demonstrated to possess good anticonvulsant properties [134]. Their substitution products have also been found a number of other pharmacological properties such as antitumor, anti-HIV and antibacterial activities [135,136,137].

He *et al.*, synthesized new 6-methyl-1-substituted-4,6-diazaspiro[2.4]heptane-5,7-diones and tested the anticonvulsant activity using the MES and *sc*-PTZ screens. Their neurotoxicity was determined by the rotarod test. The most active of the series was compound **166** (Table 8), which showed a MES ED_50_ value of 12.5 mg/kg in mice. The TD_50_ was 310 mg/kg, providing compound **166** with a PI of 24.8 in the MES test which is better than that of Phenytoin [138].

He *et al.*, investigated some new *N*-3-arylamide substituted 5,5-cyclopropanespirohydantoin derivatives synthesized and tested for anticonvulsant activity using the maximal electroshock (MES), subcutaneous pentylenetetrazole (*sc*-PTZ) screens, which are the most widely employed seizure models for early identification of candidate anticonvulsants. Their neurotoxicity was determined applying the rotorod test. The most active compound **167** showed the MES-induced seizures with ED_50_ value of 9.2 mg/kg and TD_50_ value of 421.6 mg/kg after i.p. to mice (Table 8), which provided compound **167** with a protective index (TD_50_/ED_50_) of 45.8 in the MES test [139].

Botros *et al.*, designed and synthesized new phenytoin derivatives and tested the anticonvulsant activity. Preliminary anticonvulsant screening was performed using standard MES and *sc*-PTZ screens in mice. The neurotoxicity was determined by applying the rotarod test. Among these compounds, **168** and **169** showed the highest protection (80%) in the *sc*-PTZ test at a dose of 100 mg/kg, whereas the compound **170** displayed promising anticonvulsant effect in the MES model (Table 8) [140].

Byrtus *et al.*, prepared a various of *N*-Mannich bases derived from 5-cyclopropyl-5-phenyl- and 5-cyclopropyl-5-(4-chlorophenyl)-imidazolidine-2,4-diones. The quantitative evaluation after oral administration in rats showed that the most active was compound **171** with ED_50_ values of 5.76 mg/kg (MES) and 57.31 mg/kg (*sc*-PTZ) (Table 8). Compared with the control drugs of ethosuximide and phenytoin, it was more active in the anti-convulsion assays. Additionally compound **171** with ED_50_ of 26.06 mg/kg in a psychomotor seizure test (6-Hz) in mice showed comparable activity to a new generation anticonvulsant-levetiracetam [141].

Dhanawat *et al.*, had reported a synthesis of *N*-(3)-substituted-2,4-imidazolidine diones and oxazolidinediones derivatives and tested the anticonvulsant activity using the MES test. Compounds **172**, **173**, **174** and **175** exhibited significant anticonvulsant activity as compared to the standard drug phenytoin (Table 8) [142].

Botros *et al.*, described new bivalent ligands derived from phenytoin. Initial anticonvulsant screening was performed using MES and PTZ screens in mice. Most of the test compounds were found to be effective in at least one seizure model at a dose of 100 mg/kg. Compound **176** exhibited marked anticonvulsant activity in both MES and PTZ screens (Table 8) [143].

Byrtus *et al.*, established a synthesis of *N*-Mannich from 5-cyclopropyl-5-phenyl- and 5-cyclopropyl-5-(4-chlorophenyl)-hydantoins and tested their anticonvulsant activity. The quantitative studies after oral administration to rats showed that several molecules were more potent than phenytoin and ethosuximide which were used as reference antiepileptic drugs. From the whole series, the most active was compound **177** with the ED_50_ value of 5.29 mg/kg in the MES test (Table 8) [144].

Madaiah *et al.*, demonstrated a synthesis of new 3-[(2,4-dioxo-1,3,8-triazaspiro[4,6]undec-3-yl) methyl]benzonitrile derivatives and evaluated their possible anticonvulsant activity by MES and PTZ tests. Compounds **178**, **179**, **180** and **181** showed significant and protective effect on seizure when compared with the standard drug valproate (Table 8). These compounds were found to exhibit advanced anticonvulsant activity as well as lower neurotoxicity than the reference drug [145].

Madaiah *et al.*, synthesized a series of novel 1′-[2-(difluoromethoxy)benzyl]-2′*H*,5′*H*-spiro[8-azabicyclo[3,2,1]octane-3,4′-imidazolidine]-2′,5′-dione substituted hydantoins. The novel molecules were screened for anticonvulsant activity in mice by MES and *sc*-PTZ-induced seizure tests. The neurotoxicity was assessed using the rotarod method. Compounds **182**, **183**, **184**, **185** and **186** exhibited anticonvulsant potency against MES-induced seizure and in the *sc*-PTZ model (Table 8), with lesser neurotoxicity. Some title compounds showed lesser depression on central nervous system compared to phenytoin [146].

**Table 8:** Structures of compounds **166**–**186**. 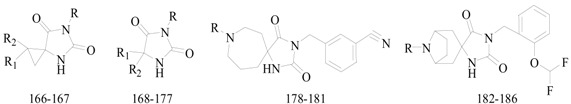

Comp. No.	R	R_1_	R_2_	Reference
**166**	-CH_3_	H	-Ph(p-SO_2_CH_3_)	[138]
**167**	-NHCOPh(*p*-CF_3_)	-CH_3_	-CH_3_	[139]
**168**	-CH_2_C(O)NNCSH	-Ph	-Ph	[140]
**169**	-CH_2_CONHNHCSNHPh(p-OCH_3_)	-Ph	-Ph	
**170**	-CH_2_CONHNHCSNHPh	-Ph	-Ph	
**171**	-H	-Ph	-C_3_H_5_	[141]
**172**	-CH_2_N(CH_2_CH_2_)_2_Ph	-Ph	-C_3_H_5_	[142]
**173**	-CH_2_CONHPh(*p*-Cl)	-Ph	-Ph	
**174**	-CH_2_CONHPh(*o*-Cl)	-Ph	-Ph	
**175**	-CH_2_CONHPh(*p*-OCH_3_)	-Ph	-Ph	
**176**	-CH_2_CON(CH_2_CH_2_)_2_Ph(*p*-NO_2_)	-Ph	-Ph	[143]
**177**	-(CH_2_)_2_O(CH_2_)_2_ N(CH_2_CH_2_)_2_Ph(*p*-Cl)	-Ph	-Ph	[144]
**178**	-SO_2_Ph(*o*-F)	—	—	[145]
**179**	-SO_2_Ph(*m*-F)			
**180**	-CO Ph(*m*-F)			
**181**	-CO Ph(*p*-F)			
**182**	-SO_2_Ph(*o*-F)			[146]
**183**	-SO_2_Ph(*m*-F)			
**184**	-SO_2_Ph(*o*-F)			
**185**	-CONHPh			
**186**	-CONHPh(*m*-CH_3_)			

## 18. The Oxime Ether Functional Group

Due to the lipophilic aryl portion facilitating penetration of the blood–brain barrier, the introduction of oxime ether groups to the compounds as a small oxygen functional group had been studied. Meanwhile, oxime ether linkages also are used as a mechanism for pro-drug generation [147].

Karakurt *et al.*, prepared oxime and oxime ether derivatives of anticonvulsant nafimidone [1-(2-naphthyl)-2-(imidozole-1-yl)ethanone] as potential anticonvulsant compounds. Most of the compounds exhibited anticonvulsant activities. Compounds **187**, **188** and **189** (salt) were found to be active at 30 mg/kg at the half-hour time point without neurotoxicity at the same dose level (Table 9). Meanwhile, these derivatives exhibited some activity against *sc*-Met as well as MES-induced seizures [148].

Karakurt *et al.*, reported synthesis of twenty-three new oxime ester derivatives of nafimidone. MES and *sc*-Met tests were employed for their anticonvulsant activities and rotarod test for neurological deficits. Compound **190** was the most active one in *sc*-Met test at all dose levels at 4 h (Table 9) [149].

Bansal *et al.*, synthesized O-alkylated derivatives of 1-(2-naphthyl)-2-(imidazol-1-yl)ethanone oxime as potential anticonvulsant compounds. Pre-treatment of mice with compounds **191** and **192** (30 mg/kg, i.p.) significantly decreased the susceptibility to PTZ-induced seizure as evidenced by delayed onset of clonus and mean onset time of extensor phase (Table 8). The treatment of mice with these compounds show equivalent protection level as compared with standard drug diazepam (0.5 mg/kg, i.p.). Anticonvulsant evaluation data showed that compounds **191** and **192** were the most active with ED_50_ values of 46.77 mg/kg and 24.41 mg/kg, respectively [150].

Karakurt *et al.*, synthesized oxime and oxime ether derivatives of [1-(2-naphthyl)-2-(1,2,4-triazol-1-yl)ethanone] as potential anticonvulsant compounds. The most active of the series was compound **193** (Table 8) [151].

**Table 9:** Structures of compounds **187**–**193**. 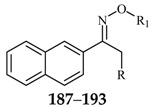

Comp. No.	R	R_1_	Reference
**187**	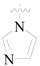	-CH_3_	[148]
**188**		-C_2_H_5_	
**189**		-CH_2_CHCH_2_	
**190**		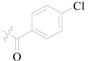	[149]
**191**		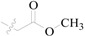	[150]
**192**		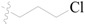	
**193**	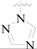	-CH_3_	[151]

## 19. The Pyridazine Functional Group

Pyridazine is a heterocyclic organic compound with the molecular formula (CH)_4_N_2_. It contains a six-membered ring with two adjacent nitrogen atoms, and is aromatic [150]. Pyridazine derivatives have various biological applications [152,153,154,155].

Guan *et al.*, synthesized a series of 6-alkoxy-[1,2,4]triazolo[4,3-*b*]pyridazine derivatives. In initial screening and quantitative evaluation, compound **194** was the most active agent, exhibiting the lowest toxicity at the same time (Table 10). In the anti-MES test, it showed ED_50_ of 17.3 mg/kg and TD_50_ of 380.3 mg/kg, and the PI of 22.0 which is much better than PI of the reference drugs [156].

**Table 10:** Structures of compounds **194**–**200**. 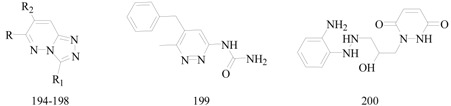

Comp. No.	R	R_1_	R_2_	Reference
**194**	-OPh(*2*,*4*-Cl_2_)	H	H	[156]
**195**	-CH_3_	H	-CH_2_Ph	[157]
**196**	-CH_3_	H	-CH_2_Ph(2,6-Cl_2_)	[158]
**197**	-CH_3_	-NH_2_	-CH_2_Ph	
**198**	-CH_3_	-NH_2_	-CH_2_Ph(2,6-Cl_2_)	

Sivakumar *et al.*, reported synthesis of 1-substituted-1,2-dihydro-pyridazine-3,6-diones as potential anticonvulsant agents. The compounds were tested *in vivo* for the anticonvulsant activity. The compound which have maximum protection against MES-induced seizures was compound **195** with ED_50_ = 44.7 mg/kg i.p. 4 h (Table 10) [157].

Moreau *et al.*, reported a synthesis of several 3-substituted pyridazines and a series of imidazo- and triazolopyridazines and tested their anticonvulsant activity against MES-induced seizures in mice. The most active derivatives, **196**, **197**, **198**, **199** and **200** with oral ED_50_ ranged from 6.2 to 22.0 mg/kg (Table 10). The compound **200** was also protective in the PTZ-induced seizure test (ED_50_ = 76 mg/kg per os) and blocked strychnine-induced tonic extensor seizures (ED_50_ = 34.5 mg/kg per os). Furthermore, derivative **200** showed anticonvulsant effects on bicuculline- and yohimbine-induced seizure tests in mice [158].

## 20. Miscellaneous Functional Groups

Sapa *et al.*, established a synthesis of some novel pyrrolidin-2-one derivatives and evaluated their possible anticonvulsant activity by MES and PTZ tests. Compound **201** significantly reduced the incidence of seizures in the MES test. The compounds **202** and **203** demonstrated activity in the PTZ-induced seizures test [159,160].

**Figure 12:**
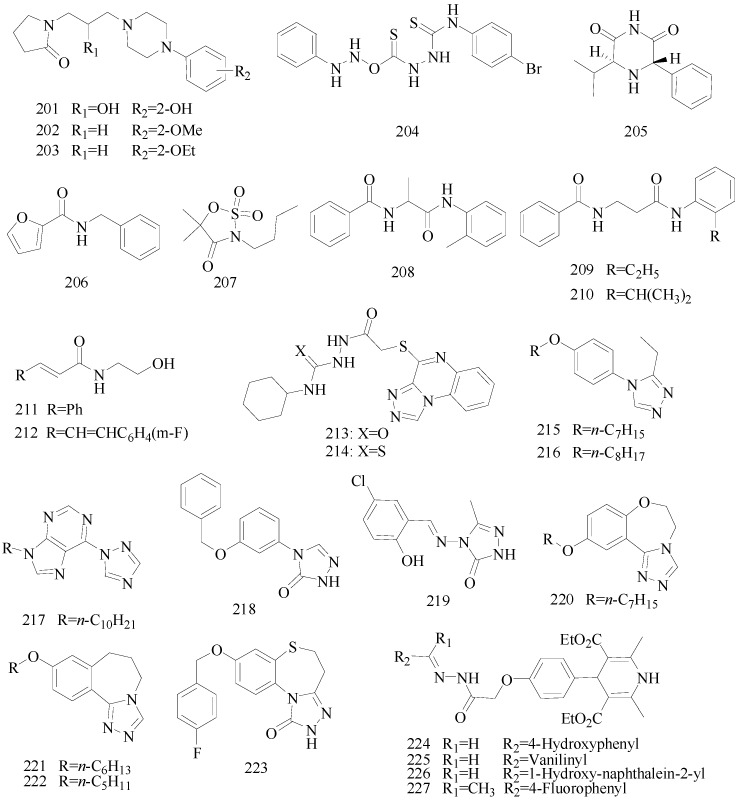
Structures of compounds **201**–**227**.

Nevagi *et al.*, demonstrated synthesis of novel thiosemicarbazide derivatives and evaluated their anticonvulsant activity and neurotoxicity. Amongst all the synthesized compounds, compound **204** is a broad-spectrum anticonvulsant agent since it was active in both MES- and PTZ-induced seizure models with no neurotoxicity (Figure 12) [161].

Dawidowski *et al.*, synthesized a series of novel diastereomerically pure, monocyclic 2,6-DKP derivatives using a diastereoselective method. In the MES test, some of them showed good or weak antiepileptic activities, however, there was no active compound in the *sc*-Met screen. The most promising compound **205** exhibited notable action in the 6 Hz test (Figure 12) [162].

Strupińska *et al.*, synthesized a series of benzylamides of isocyclic and heterocyclic acids and tested their anticonvulsant activity. Nearly all synthesized heterocyclic acid derivatives showed anticonvulsant activity. Compound **206** appeared the most promising (Figure 12). It showed in minimal clonic seizure (6 Hz) test (ASP) in rats after i.p. administration: MES ED_50_ = 36.5 mg/kg, TOX TD_50_ = 269.75 mg/kg, and PI = 7.39 [163].

Pastore *et al.*, synthesized novel *N*-derivative-1,2,3-oxathia-zolidine-4-one-2,2-dioxides heterocycles, bioisosteres of trimethadione (TMD, oxazolidine-2,4-dione) and phenytoin. Anticonvulsant screening was performed in mice after intraperitoneal administration in the MES test and *sc*-PTZ test. Compound **207** (Figure 12), the most active drug obtained, with an ED_50_ of 60 μg/kg was 10,000 times more active than TMD, the reference compound in this work, and 90 times more active than valproic acid, an anticonvulsant drug presently in use in the clinic [164].

Uysal *et al.*, designed and synthesized sixteen 2/3-benzoylaminopropionanilide derivatives. The anticonvulsant activity profile of the synthesized compounds was determined by MES and *sc*-Met seizure tests. In the rotarod test, all of them exhibited no toxicity to the nervous system. Compounds **208**, **209** and **210** were found to be more potent in the MES or *sc*-Met tests (Figure 12). Those compounds have emerged as lead compounds for future investigations [165].

Guan *et al.*, prepared a variety of *N*-(2-hydroxyethyl)cinnamamide derivatives and screened their anticonvulsant activities by the MES test and their neurotoxicity was evaluated by the rotarod test. In the anti-MES potency test, compounds **211** and **212** exhibited ED_50_ dose of 17.7 and 17.0 mg/kg, respectively (Figure 12), and TD_50_ dose of 154.9 and 211.1 mg/kg, respectively, resulting in a PI of 8.8 and 12.4, respectively, which were much greater than the PI of the market antiepileptic drug carbamazepine [166].

Alswah *et al.*, reported synthesis of some [1,2,4]triazolo[4,3-*a*]quinoxaline derivatives as novel anticonvulsant agents. The anticonvulsant evaluation was used metrazol-induced convulsion model and phenobarbitone sodium was as a standard. Among this set of tested compounds, two of them (**213** and **214**) showed the best anticonvulsant activities (Figure 12) [167].

Chen *et al.*, reported synthesis of 4-(4-alkoxylphenyl)-3-ethyl-4*H*-1,2,4-triazole derivatives. Their anticonvulsant activities were evaluated by the MES test and their neurotoxicity was evaluated by the rotarod test. MES test showed that compound **215** was found to be the most potent with ED_50_ value of 8.3 mg/kg and PI value of 5.5, but compound **216** exhibited better PI value of 9.3 (Figure 12), which was much greater than PI value of the prototype drug phenytoin [168].

Wang *et al.*, synthesized a series of new purines containing triazole and other heterocycle substituents and evaluated their preliminary anticonvulsant activity and neurotoxicity by using the MES, *sc*-PTZ and rotarod tests. Among the compounds studied, compound **217** was the most potent compound, with a ED_50_ of 23.4 mg/kg and a high protective index of more than 25.6 after intraperitoneal administration in mice (Figure 12). Compound **217** showed significant oral activity against MES-induced seizures in mice, with an ED_50_ of 39.4 mg/kg and a PI above 31.6 [169].

Shu *et al.*, reported synthesis of 4-(3-alkoxy-phenyl)-2,4-dihydro-[1,2,4]triazol-3-ones. All target compounds exhibited anticonvulsant activity of varying degrees in the maximal electroshock test. Compound **218** was the most promising compound with an ED_50_ value of 30.5 mg/kg and a PI of 18.63 (Figure 12), showing a higher safety than the standard drug carbamazepine (PI = 6.45). In addition, the potency of compound **218** against seizures induced by pentylenetetrazole and 3-mercaptopropionic acid suggested its broad-spectrum activity [170].

Kahveci *et al.*, designed and synthesized a series of new 1,2,4-triazole-3-one derivatives bearing the salicyl moiety. The anticonvulsant activities of all compounds were evaluated by the Anticonvulsant Screening Program of the U.S. National Institutes of Health. The most active compound **219** showed significant anticonvulsant activity with an ED_50_ of 81.1 mg/kg at an approximate TPE (time of peak effect) of 1 h (Figure 12) [171].

Deng *et al.*, reported a synthesis of 10-alkoxy-5,6-dihydrotriazolo[4,3-*d*]benzo[*f*][1,4] oxazepine derivatives and screened their anticonvulsant activities by the MES test and their neurotoxicity was evaluated by the rotarod test. In the MES test, compound **220** was found to possess better anticonvulsant activity and higher safety than market drugs carbamazepine and phenytoin with an ED_50_ value of 6.9 mg/kg a PI value of 9.5 (Figure 12) [172].

Piao *et al.*, reported a novel series of 9-alkoxy-6,7-dihydro-5*H*-benzo[*c*][1,2,4]triazolo[4,3-*a*] azepine derivatives and screened their anticonvulsant activity by the MES test and the *sc*-PTZ test. The results revealed that all of the compounds exhibited anticonvulsant activity, compound **221** was found to possess the most potent anticonvulsant activity in the anti-MES potency test (Figure 12), it had a ED_50_ value of 12.3 mg/kg, a TD_50_ value of 73.5 mg/kg, and a PI of 6.0, which was slightly lower than the PI of the prototype drug carbamazepine (ED_50_ = 8.8, PI = 8.1). In the *sc*-PTZ test, compound **222** was the most active, with an ED_50_ value of 19.8 mg/kg, a TD_50_ value of 80.8 mg/kg and a PI value of 4.1, which are greatly higher than that of carbamazepine (ED_50_ > 100, PI < 0.72) [173].

Deng *et al.*, synthesized two series of 8-alkoxy-4,5-dihydrobenzo[*b*][1,2,4]triazolo[4,3-*d*] [1,4]thiazepine derivatives. All of the prepared compounds were effective in the MES screens, among which, compound **223** was considered as the most promising one with an ED_50_ value of 26.3 mg/kg and a superior PI value of 12.6 (Figure 12). The potency of compound **223** against seizures induced by pentylenetetrazole, 3-mercaptopropionic acid and bicuculline was great too [174].

Ulloora *et al.*, synthesized new substituted 1,4-dihydropyridin-4-yl-phenoxyacetohydrazones. The final compounds were screened for their *in vivo* anticonvulsant activity by MES, *sc*-PTZ and 6 Hz methods. The active compounds, **224**, **225**, **226** and **227** exhibited their activities at 4 h after i.p. injection with 100 mg/kg (Figure 12). All these tested compounds exhibited activity in 6 Hz method within 1 h [175].

**Figure 13:**
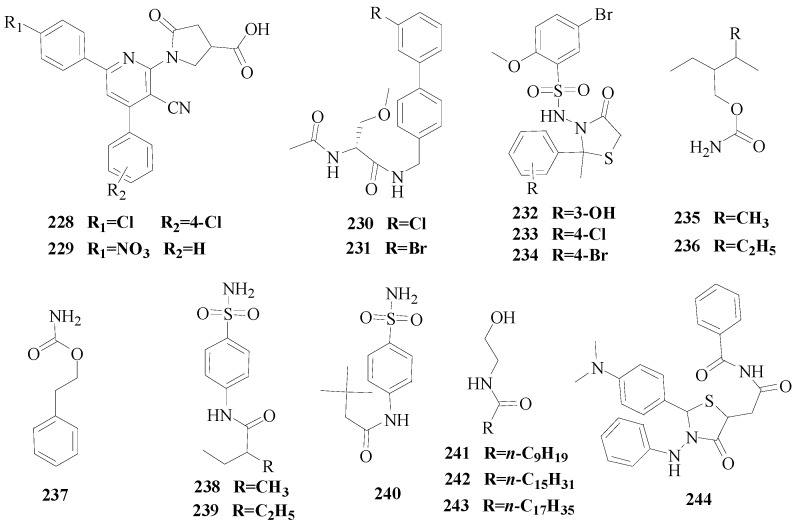
Structures of compounds **228**–**244**.

Siddiqui *et al.*, reported synthesis of various 1-[6-(4-substituted phenyl)-3-cyano-4-(substituted phenyl)-pyridin-2-yl]-5-oxopyrrolidine-3-carboxylic acids. Their *in vivo* anticonvulsant evaluation was performed by MES and *sc*-PTZ tests. Compounds **228** and **229** displayed comparable anticonvulsant activity to the standard drugs with ED_50_ values of 13.4 and 18.6 mg/kg in electroshock screen, respectively (Figure 13). The compounds **228** and **229** were also found to have encouraging anticonvulsant activity (ED_50_ = 86.1 and 271.6 mg/kg, respectively) in *sc*-PTZ screen. Interestingly, they did not show any sign of motor impairment at the maximum dose administered and were not toxic to the liver [176].

Lee *et al.*, prepared 13 derivatives of *N*-(biphenyl-4′-yl)methyl-(*R*)-2-acetamido-3-methoxy-propionamide that were tested for anticonvulsant activity at the Anticonvulsant Screening Program (ASP) of the National Institute of Neurological Disorders and Stroke (NINDS) of the U.S. National Institutes of Health. The excellent activities in the MES test (mice, i.p.) of the compound **230** and **231** (ED_50_ = 9.8 and 12 mg/kg, respectively) coupled with their low neurotoxicities (TD_50_ = 74 and 86 mg/kg, respectively) provided compounds with notably higher PI (7.6 and 7.2, respectively) (Figure 13) [177].

Siddiqui *et al.*, prepared a series of 4-thiazolidinones bearing a sulfonamide group and tested their anticonvulsant activity utilizing MES and *sc*-PTZ animal models. Compounds **232**, **233** and **234** displayed promising activity and could be considered as leads for further investigations (Figure 13) [178].

Hen *et al.*, synthesized a novel class of 19 carbamates and evaluated their anticonvulsant activity in the rat MES and *sc*-Met seizure tests and pilocarpine-induced status epilepticus (SE) model. The carbamates **235** (MES ED_50_ = 64 mg/kg), **236** (MES ED_50_ = 52 mg/kg) and **237** (MES ED_50_ = 16 mg/kg) offered an optimal anticonvulsant efficacy and safety profile and consequently are potential candidates for further development as new AEDs (Figure 13) [179].

Hen *et al.*, synthesized a novel class of aromatic amides composed of phenylacetic acid or branched aliphatic carboxylic acids, with five to nine carbons in their carboxylic moiety, and aminobenzenesulfonamide. The final compounds were screened for their anticonvulsant activity by MES and *sc*-Met tests. The amides **238**, **239** and **240** were the most potent compounds possessing MES-ED_50_ values of 7.6, 9.9, and 9.4 mg/kg and remarkable PI values of 65.7, 50.5, and 53.2, respectively (Figure 13) [180].

Guan *et al.*, demonstrated a synthesis of novel series of *N*-(2-hydroxyethyl)amide derivatives and screened their anticonvulsant activities by the MES test, and their neurotoxicity was evaluated by the rotarod test. The MES test showed that compounds **241**, **242** and **243** were found to show a better anticonvulsant activity and also had lower toxicity than the market anti-epileptic drug valproate (Figure 13). 

In the anti-MES potency test, these compounds exhibited ED_50_ doses of 22.0, 23.3, 20.5 mg/kg, respectively, and TD_50_ doses of 599.8, >1000, >1000 mg/kg, respectively, resulting in a PI of 27.5, >42.9, >48.8, respectively, which are much higher than valproate (PI = 1.6) [181].

Senthilraja *et al.*, synthesized a new series of 2-(4-dimethylaminophenyl)-3-substituted thiazolidin-4-one-5-yl-acetyl acetamides/benzamides. The title compounds were investigated for their anticonvulsant activities, among the test compounds, compound **244** emerged as the most active compound of the series and as moderately more potent than the reference standard diazepam (Figure 13) [182].

## 21. Conclusions

All in all, based on our laboratory work and the recent literature, this review summarized some significant anticonvulsant compounds which are classified by functional groups and according to data obtained by studies designed in animal models. This review illustrates the various attempts made to discover and develop antiepileptic compounds with more effective and selective effects, and reduced secondary actions. The extensive work reviewed here may represent a starting point to allow a better understanding of antiepileptic therapeutic developments as well as to suggest ideas on design and synthesis of novel antiepileptic compounds.
